# Determination of Residual Triflumezopyrim Insecticide in Agricultural Products through a Modified QuEChERS Method

**DOI:** 10.3390/foods10092090

**Published:** 2021-09-03

**Authors:** Sung-Min Cho, Han-Sol Lee, Ji-Su Park, Su-Jung Lee, Hye-Sun Shin, Yun-Mi Chung, Ha-Na Choi, Yong-Hyun Jung, Jae-Ho Oh, Sang-Soon Yun

**Affiliations:** 1 Food Safety Evaluation Department, Pesticide and Veterinary Drug Residues Division, National Institute of Food and Drug Safety Evaluation, Ministry of Food and Drug Safety, Cheongju 28159, Korea; smcho.0101@gmail.com (S.-M.C.); leehs3029@korea.kr (H.-S.L.); jeesoo0320@korea.kr (J.-S.P.); bplsj@korea.kr (S.-J.L.); hyesun0714@korea.kr (H.-S.S.); jyh311@korea.kr (Y.-H.J.); chopin68@korea.kr (J.-H.O.); 2 Department of Integrated Biomedical and Life Science, Graduate School, Korea University, Seoul 02841, Korea; 3 Hazardous Substances Analysis Division, Gwangju Regional Food and Drug Administration, Gwangju 61012, Korea; gd96@korea.kr (Y.-M.C.); chlgkskgg@korea.kr (H.-N.C.)

**Keywords:** triflumezopyrim, pesticide residue, food, QuEChERS, maximum residue limit

## Abstract

A rapid and simple analytical method for triflumezopyrim, a new class of mesoionic insecticides and commercialized molecules from DuPont, was developed with a modified QuEChERS method. The pH adjustment was used to improve the extraction efficiency of acetonitrile solvent, and dispersive solid-phase extraction was employed for the clean-up process. The five selected food commodities were used to verify the present optimized method, which displayed good linearity with an excellent correlation coefficient (*R*^2^ = 0.9992–0.9998) in the 0.003–0.30 mg/kg calibration range. The method limits of detection (LOD) and quantification (LOQ) were determined to be a value of 0.003 and 0.01 mg/kg, respectively. The mean recovery for the triflumezopyrim was in the 89.7–104.3% range. The relative standard deviations were ≤9.8% for intra- (*n* = 5) and inter-day (*n* = 15) precisions at concentrations of 0.01, 0.1, and 0.5 mg/kg in the five representative samples. The matrix effect has been calculated to confirm the effect during ionization of the analyte in the UPLC-MS/MS. The matrix effects of the instrumental analysis showed that triflumezopyrim was less susceptible to matrices. The proposed analytical method in this study has effectively improved the accuracy, selectivity, and sensitivity for the determination of triflumezopyrim in agricultural commodities; therefore, it can serve as a reference method for the establishment of maximum residue limits (MRLs).

## 1. Introduction

Triflumezopyrim is a novel class of mesoionic chemical pesticides with reasonable insecticidal activity and has shown high efficiency at a low dosage for outstanding control of susceptible and resistance to a wide spectrum of hopper species in rice throughout the Asia region [1,2,3]. According to the literature [4,5,6], the mode of action of triflumezopyrim is known to lead to the over-excitation of the insect nervous system through binding to and inhibiting the orthosteric site of its nicotinic acetylcholine receptor (nAChR). From the successful discovery of triflumezopyrim and commercialization by DuPont Crop Protection, it is important to establish a new standard method for the determination of triflumezopyrim residue in foods, assess the risk of toxicological effects, and set maximum residue limits (MRLs) to protect public health on designing safety guidelines and regulations.

According to the risk assessment report of the Food Safety Commission of Japan (FSCJ), the no-observed-adverse-effect level (NOAEL) for the triflumezopyrim was determined to be 3.23 mg/kg bw/day from a two-year combined chronic toxicity and carcinogenicity study in rats and the acceptable daily intake (ADI) was set at 0.032 mg/kg bw/day by considering a safety factor of 100 relatives to the NOAEL [7]. In the joint FAO/WHO meeting on pesticide residues (JMPR), the NOAEL for triflumezopyrim (400 ppm, equal to 12.2 mg/kg bw per day) was obtained from a 90-day dog study which considered the effects of body weight and the secondary effects such as the lymphoid depletion in the thymus [8]. The ADI was estimated as 0.12 mg/kg bw/day by considering a 100-fold safety factor. In the Codex Alimentarius Commission (CAC), the definition of the residual triflumezopyrim considers the parent compound alone, and the MRLs were set at 0.01–0.2 mg/kg for rice [9]. The MRLs set by the Japan Food Chemical Research Foundation (JFCRF) and the United States Environmental Protection Agency (EPA) were 0.01 mg/kg for rice and 0.4–1.0 mg/kg for two related items.

In South Korea, the application for the registration of triflumezopyrim in rice was submitted to the Rural Development Administration, and the Ministry of Food and Drug Safety of Korea (MFDS) is expected to set the MRLs based on the present study. The Korean Positive List System (PLS) was also adapted to tree nuts/oil seeds and tropical fruit since December 2016 (MFDS notification No. 2015-78) and was expanded to all agricultural products sold and marketed in Korea from January 2019. In addition, the standard MRLs are applied for the pesticides that are detected in domestic or imported agricultural products in Korea. In cases where the standard of MRLs is not set, the limit of quantification (LOQ) for the pesticide must be less than or equal to 0.01 mg/kg to ensure safe pesticide management. In this context, the establishment of a specific and sensitive method is highly necessary for reliable monitoring of the residual triflumezopyrim in agricultural products.

Pesticides are among the most toxic substances contaminating the environment. They are particularly dangerous in fruit and vegetables, by which people are exposed. It is crucial to monitor pesticide residues in fruit and vegetables using available analytical methods [10,11,12]. Since triflumezopyrim is a newly registered pesticide in 2019, there is still no standardized analysis method in Korea yet. It is necessary that the development of a sensitive analytical method allows determining the triflumezopyrim in foods. It has been reported that the determination of pesticide residues in foods is a difficult challenge because of the small quantities of analytes and large amounts of interfering substances that can be co-extracted with analytes and affect the results of an analysis [13].

The determination of a target compound from a complex matrix requires its extraction and purification for facilitating analysis. However, conventional methods for extraction, such as the liquid-liquid and soxhlet extractions, and the related approaches are time-consuming and require large amounts of organic solvents. After extraction, purification, such as solid-phase extraction or liquid-liquid extraction, is used to extract the target compounds from aqueous samples. However, two steps are typically laborious, time-consuming and high solvent consumption, which leads to reduced recovery. To improve the efficiency for the determination of pesticide residues in various matrices, the QuEChERS (quick, easy, cheap, effective, rugged, and safe) method has been applied for the extraction and clean-up [14,15]. For extraction, organic solvent and salts are used and then followed by purification with dispersive solid-phase extraction (dSPE). This technology is rapid, simple and requires low solvent consumption, which represents a significant advantage in environmental and economic aspects [16,17,18]. In conjunction, high-performance liquid chromatography-tandem mass spectrometry (HPLC-MS/MS) has been used to sensitively and selectively detect multiple pesticides in fruit, vegetables, cereal, and related foods [19]. Very recently, an uptake ability and distribution of triflumezopyrim in rice, water, and soil has been reported using HPLC-MS/MS analysis by simulating the application of triflumezopyrim on rice [20]. Compared to the conventional LC-MS/MS method, the ultra-performance liquid chromatography (UPLC)-MS/MS method could offer many advantages of improved resolution and higher sensitivity, as well as shorter analysis time since this technique use column packed with smaller particle size and is operated at a higher flow rate than those of LC-MS/MS technique [21,22]. The aim of this study is to develop a government-sponsored official analysis method for the determination of potential triflumezopyrim residue in agricultural products to enable efficient monitoring and safety management of the domestic and imported ones. Although several studies have investigated the toxicity of triflumezopyrim, to the best of our knowledge, no studies have been reported yet on the development and validation of an analytical method for the determination of triflumezopyrim residue in naturally-grown food matrices by optimizing a modified QuEChERS method with UPLC-MS/MS analysis. Furthermore, we also applied to a variety of selected agricultural samples to future monitor pesticide residues.

## 2. Materials and Methods

### 2.1. Chemicals and Reagents

Triflumezopyrim (DPX-RAB55, purity 99.4%) standard was supplied by DuPont Crop (Newark, DE, USA). HPLC grade acetonitrile was purchased from Merck (Darmstadt, Germany). Sodium chloride (NaCl), anhydrous magnesium sulfate (MgSO_4_), and formic acid (reagent grade, ≥95%) were purchased from Sigma Aldrich (St. Louis, MO, USA). The graphitized carbon black (GCB) and C_18_ (Octadecyl) were purchased from Waters (Leinster, Ireland). The syringe filters polytetrafluoroethylene (PTFE, 0.2 μm pore size, 13 mm diameter) were purchased from Teknokroma (Barcelona, Spain).

Generally, the use of representative commodities is important for the validation of an analytical method. For this purpose, five agricultural products, including mandarin, potato, green pepper, hulled rice, and soybean, were selected from the representative species, which are classified according to the Codex guidelines on good laboratory practice in pesticide residue analysis (Table S1) [23]. Those five agricultural products were purchased from the local market, in which the target pesticide of triflumezopyrim was not detected. A total of 50 samples of mandarin, potato, green pepper, hulled rice, and soybean were purchased from the Hyundai department store in the city of Cheongju (population of 831,600). The agricultural products selected in this study were organically produced in different regions of South Korea and also were intended for domestic consumption. The samples (at least 1 kg per commodity) were chopped and homogenized by the MFDS sample preparation procedure and kept in the dark below −50 °C until further study.

### 2.2. Sample Preparation

Each agricultural product (5 g) was weighed into each conical tube, and 15 mL of acetonitrile was added. After shaking the sample and the solvent for 10 min, the mixture was adjusted using a pH meter. The pH of the extracts was adjusted to 5 using formic acid, and the samples were shaken for 10 min. After the extraction, 1 g of NaCl and 3 g of anhydrous MgSO_4_ were added to the mixture of the extracts, which was shaken for 1 min, followed by centrifugation at 7000× *g* for 10 min. Subsequently, 5 mL of the supernatant of the extracts was transferred into a 15 mL conical tube containing an adsorbent mixture comprising 250 mg of PSA, 250 mg of C_18_, and 30 mg of GCB. The tubes were shaken for 1 min and centrifuged at 7000× *g* for 5 min. The supernatant layers (1 mL) were filtered through a 0.22 μm PTFE membrane into LC vials for LC-MS/MS analysis (Figure S1).

### 2.3. UPLC-MS/MS Analysis

The analysis of the triflumezopyrim was conducted on a Nexera X2 UHPLC system coupled with an 8060 triple quadrupole mass spectrometer (Shimadzu, Kyoto, Japan). The labsolution software was used for instrument control and data acquisition, and a Waters XBridge C_18_ column (100 mm I.D. × 2.1 mm L., 3.5 μm particle size) was employed and the column temperature was maintained at 40 °C. The mobile phase consisted of (A) 0.1% formic acid in acetonitrile and (B) 0.1% formic acid in water. The separation was performed in the gradient mode: 0.0–1.0 min (10% A), 1.1–4.0 min (30% A), 4.1–7.0 min (70% A), 7.1–8.0 min (30% A), and 8.1–10.0 min (10% A). The flow rate was set to 0.3 mL/min, and the injection volume was 2 μL. The UPLC-MS/MS was operated in the positive electrospray ionization (ESI^+^) and multiple reaction monitoring (MRM) modes, and the optimized precursor/product ion was selected by adjusting the collision energy (CE) in the collision cell. Nitrogen was used as the nebulizer gas, and argon was used as the collision gas. The interface temperature was maintained at 150 °C, and the nebulizing gas flow rate was set to 3.0 L/min. The analytical operating conditions for the determination of triflumezopyrim are summarized in Table S2. The pesticide residues analysis in food was performed according to the identification criteria (SANTE/12682/2019) established in the EU [24].

### 2.4. Method Validation

Triflumezopyrim (10.06 mg) stock solution was prepared by its dissolution in acetonitrile (1000 mg/L). The standard and working solutions were prepared for the construction of calibration curves and recovery tests and were stored in the dark at 4 °C. For the preparation of the matrix-matched standard solution, the working solution was added to the extracts (≥90% *v*/*v*) of the non-spiked samples at the following concentration levels: 0.003, 0.006. 0.012, 0.03, 0.06, 0.15 and 0.30 µg/mL (Table S3).

The developed method was validated for linearity, LOD, LOQ, accuracy, and precision. Selectivity was confirmed by comparing the chromatograms of non-spiked samples and spiked samples. Linearity was evaluated by the matrix-matched calibration curve using the standard solution (0.003–0.3 μg/mL) diluted with the extract of non-spiked samples (≥90%, *v*/*v*). The correlation coefficient (*R*^2^) and linear regression equations were calculated using the calibration curve. The matrix-effect (ME) (%) was evaluated by comparing the slope ratio of the matrix-matched calibration curve with that of the solvent-based calibration curve. The method’s LOD and LOQ, evaluated as the S/N (signal-to-noise ratio), were determined using a minimum concentration in each matrix. For precision and accuracy, intra-day (repeatability) and inter-day precision (reproducibility) were evaluated by analyzing the sample on the same day and during 15 consecutive days, respectively. The recovery and relative standard deviation (RSD) were confirmed using agricultural samples spiked with different concentrations at 1× LOQ, 10× LOQ, and 50× LOQ (*n* = 5), respectively.

## 3. Results and Discussion

### 3.1. Optimization of Instrument Conditions and Chromatograms

Triflumezopyrim consists of both polar and nonpolar functionalities and has a Log P_ow_ of 1.26 and vapor pressure of 2.65 × 10^−8^ Pa at 25 °C. While UPLC-MS/MS was suitable for its detection, GC was not suitable for its analysis due to its low vapor pressure and poor heat stability. According to PLS, the LOQ should not exceed the 0.01 mg/kg level, which was satisfactory with the use of the operating LC-MS/MS conditions. Furthermore, the UPLC-MS/MS analysis also displayed high selectivity and sensitivity at low concentration levels.

The standard solution of the triflumezopyrim (0.1 μg/mL) was directly infused into the mass spectrometer in the MRM mode for optimizing the MS/MS parameters. Triflumezopyrim was detected in the positive and negative ion modes, and its intensity was higher in the positive ion mode than in the negative one. The mass of triflumezopyrim is 398.09 g/mol, and the protonated molecular ion [M+H]^+^ was confirmed with the appearance of the 399.00 *m*/*z* as the precursor ion. The optimal setting for the generation of the precursor/production ion was chosen by adjusting the CE. The most abundant ion in the spectrum was 278 *m*/*z,* which was used for quantification. The lesser abundant ions of 121 *m*/*z* and 306 *m*/*z* were used for qualification. The optimal analysis conditions are summarized in Table 1.

The LC column, mobile phase, and flow rate were optimized to choose the suitable peak shape and retention time. The separation was conducted with a reverse-phase column (XBridge C_18_, 2.1 mm I.D. × 100 mm L., 3.5 µm) employing a mobile phase gradient.

The use of 0.1% formic acid in acetonitrile–water as the gradient provided a sharper peak shape than that realized with the use of acetonitrile–water in the absence of formic acid. The formic acid additive served as a protonation enhancer in the mobile phase and also improved the ion response of [M+H]^+^, the separation and sensitivity as well. Further, the shoulder peak disappeared when the composition of the mobile phase was 70% of 0.1% formic acid in acetonitrile rather than 90%. Overall, a separation of 5.3 min was obtained, and the absence of interference peaks was confirmed, as shown in Figure S2.

### 3.2. Optimization of Extraction and Clean-Up

A method called QuEChERS, developed initially by Anastassiades for the determination of pesticide residues in food, has been employed in various food matrices. After initial single-phase extraction of the sample with acetonitrile, clean-up is performed by a dispersive solid-phase extraction using PSA, etc., followed by quantitative and confirmatory analysis with GC-MS/MS and LC-MS/MS. The two QuEChERS versions available for this purpose are the AOAC Official Method 2007.01, which uses acetate buffering, and the European Committee for Standardization (CEN) Standard Method EN 15662, which uses citrate buffering [25]. While we evaluated both these QuEChERS versions to detect triflumezopyrim, the result of the recovery was 64.6–70.0% for mandarin.

Water-miscible solvents (acetone and acetonitrile) are used for the extraction of polar and nonpolar pesticide residues from non-fatty foods. Considering the physiochemical characteristics of triflumezopyrim, the samples were extracted with polar organic solvents such as acetonitrile, methanol, and acetone. Since the solubility of triflumeopyrim is 65.9 and 71.9 g/L in acetonitrile and acetone, respectively, these solvents were suitable for the extraction from the aqueous matrix. The extraction recovery of mandarin in acetonitrile was 80.0 ± 1.6%, compared to the 70.3 ± 1.2% in methanol. Acetonitrile is known for its high extraction efficiency in the presence of polar interferences from the matrix such as soybeans, whereas acetone is suitable for the extraction of nonpolar components such as pigments [26].

**Figure 1:**
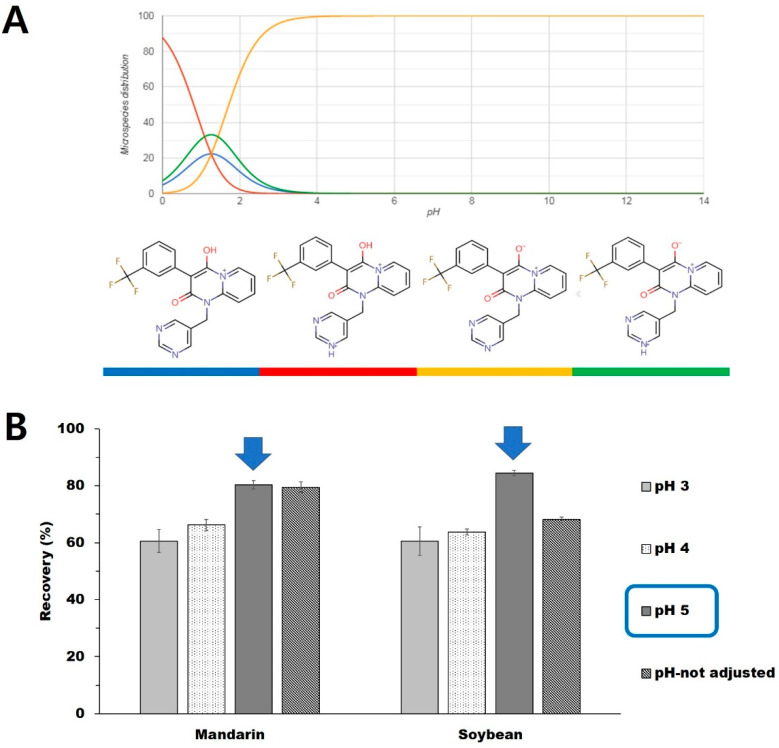
(**A**) pKa graph of triflumezopyrim and its molecular form at different pH value and (**B**) the effect of pH on extraction recovery in mandarin and soybean food matrices.

Therefore, acetonitrile was a suitable extraction solvent for triflumezopyrim since it minimizes the extraction of components such as chlorophyll. Triflumezopyrim is ion-suppressed at pH 1, and its microspecies distribution is low at 20%, which should theoretically allow their partial extraction as non-ionized lipid-soluble components into nonpolar organic solvents at a pH as shown in Figure 1A. We investigated the extraction efficiencies from the mandarin and soybean samples at various pHs that were adjusted using formic acid. The extraction recovery with and without pH adjustment to 5 was similar at 79.5–80.3% for mandarin since the original pH of mandarin is close to 5. However, for soybean, the pH adjustment to 5 afforded good extraction efficiency (84.5%) since the original pH of soybean was about 7 (68.1%) (Figure 1B).

In order to extract residual pesticides from the dry sample, the process of wetting with distilled water is essential. In addition, when 10 mL of solvent was added to 5 g of soybean sample, the mixture became too stiff to extract the residue evenly. Since the protein in the soybean containing fat acts as an emulsifier, the separation of the organic solvent layer from the water was not clear, and the amount of the extracted solution was reduced [27,28,29]. The 5 g of sample was then extracted with 15 mL of solvent.

The extraction into the organic solvent from the aqueous matrix with the addition of NaCl (salting-out) improves the extraction efficiency and minimizes the transfer of the unwanted matrix components into the organic phase due to the increased ionic strength of the aqueous matrix [30,31]. We also employed drying agents such as MgSO_4_ to remove the water from the organic extracts (Table S4). However, the organic solvent layer containing the extracted pesticide retained residual water, which affected recovery. Next, we conducted further purification by SPE to obtain the final extract, which had less interference.

Fruit and vegetables consist of an extensive variety of components, such as pigments, sugars, and other substances. To obtain good recovery results, the removal of interference was necessary for the clean-up procedure. For the optimization of the clean-up, the solvent and solvent ratio for the SPE with the cartridge (Florisil^®^, Silica, and NH_2_) were investigated using dichloromethane, n-hexane, ethyl acetate, and acetone [32,33,34]. While the mixture of dichloromethane and acetone afforded good recoveries ranging from 91.2–93.4% with the NH_2_ cartridge, the method could not be used for all samples, as the recoveries were not good.

Dispersive solid-phase extraction (d-SPE) allows the use of varying amounts and/or mixtures of the sorbent [35,36,37]. We studied the use of several types of sorbents as alternatives in the clean-up step. The primary secondary amine (PSA) sorbent has a WAX function and is highly efficient for the removal of polar compounds such as sugars and fatty acids from a nonpolar matrix. On the other hand, octadecylsilane (C_18_) is efficient for removing grease. In contrast, GCB removes pigments, including chlorophyll and carotenoids from vegetable and other extracts, and is suitable for the extraction of highly polar groups via hydrogen-bond formation due to its large surface area.

**Figure 2:**
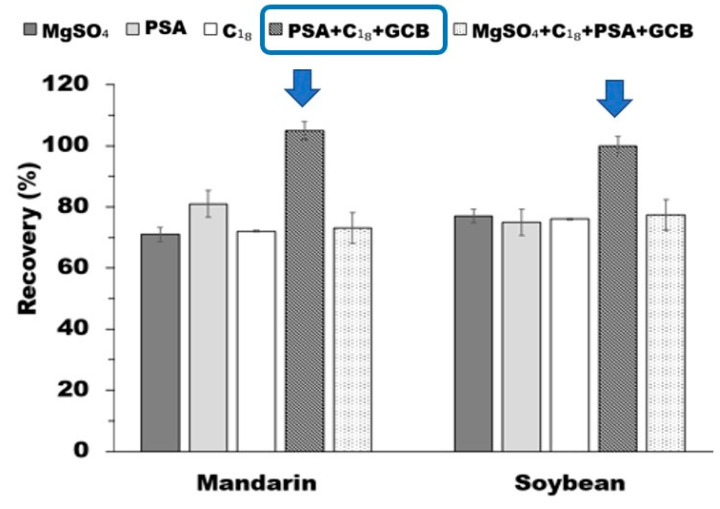
Recovery with different kinds of sorbents for triflumezopyrim adsorption in samples.

The use of these four types of adsorbents (MgSO_4_, PSA, C_18_, and GCB) was investigated in the purification step for the mandarin and soybean samples. While MgSO_4_ furnished low recovery (51.5–77.0%), PSA and C_18_ afforded recoveries in the 75.0–80.0% range. Mixing with GCB was more effective than using C_18_ and PSA alone (An et al., 2018). When the mixture of these four adsorbents (MgSO_4_ + PSA + C_18_ + GCB) was used, the recovery was 26.5% in the blank and 73.1–77.4% in the samples. When other mixtures of the adsorbents (PSA + C_18_ + GCB) were applied in the blank and samples, the recovery was satisfactory (90.0–105.0%). Therefore, 250 mg of PSA, 250 mg of C_18_, and 30 mg of GCB were used for the purification of the triflumezopyrim from the agricultural products, as shown in Figure 2 and Table S5. The modified QuEChERS method in this study, such as pH control in the extraction step and combination of d-SPE with GCB, takes advantage of the improvement of the analytical method and validation for complex food matrices, including pigments and/or fat in the lower/higher range of pH, that usually require high resolution for identifying and quantifying of pesticide residues. It can be also useful to develop multi-pesticide methods in the future.

### 3.3. Method Validation

The selectivity was measured by confirming the absence of the interfering peaks from the extracts of the blank samples at the same retention times as that of the product ion by considering its peak area. The linearity of the matrix-matched calibration curves (mandarin, potato, green pepper, hulled rice, and soybean) was evaluated with different concentrations ranging from 0.003–0.30 mg/kg. The correlation coefficient (*R*^2^) of matrix-matched calibration curves was over 0.9992 (Figure S3), which showcases the excellent selectivity and linearity of the method.

The matrix-effect profiles can be used to visualize the effect of the sample matrix on the data signals occurring in a chromatogram. The matrix-effect is the effect between the food sample and the target analyte in the ionization process in the detector of LC-MS/MS. In order to reduce the effect, various studies have been attempted, and matrix-matched calibration was a simple way to offset the matrix-effect [38]. The matrix-effect was evaluated by comparing the slope ratio of the standard solution calibration curve with that of the matrix-matched calibration curve (blank extracts) at seven concentration levels. The matrix-effect can be calculated using the equation: %ME = ((slope of matrix-matched calibration curve/slope of solvent-based calibration curve) − 1) × 100 [39]. Signal suppression and enhancement are affected by several factors, such as background noise, sample contamination, mobile phase composition, etc., and the influence of the ionization yield of the analytes. The matrix enhancement and suppression occur when ME > 0%, and ME < 0%, respectively. Further, during matrix enhancement, |ME| < 20% is considered as the lack of matrix effect, 20% < |ME| < 50% as medium matrix-effect, and |ME| > 50% as a strong matrix-effect. Triflumezopyrim showed mild signal suppression as |ME| ranged from 4.8 to 12.5, which indicated the lack of obvious matrix effects in all matrices (Table 2).

In this study, the calibration for triflumezopyrim was performed using external matrix-matched standards to eliminate the matrix-effect and obtain more accurate quantification in all samples. The method LOD and LOQ determined using the signal-to-noise ratio (S/N) were 0.003 and 0.01 mg/kg, respectively. The recovery test was conducted with three spiking levels (1, 10, and 50-fold of LOQ) to confirm the accuracy of the optimized method for all analytes.

**Figure 3:**
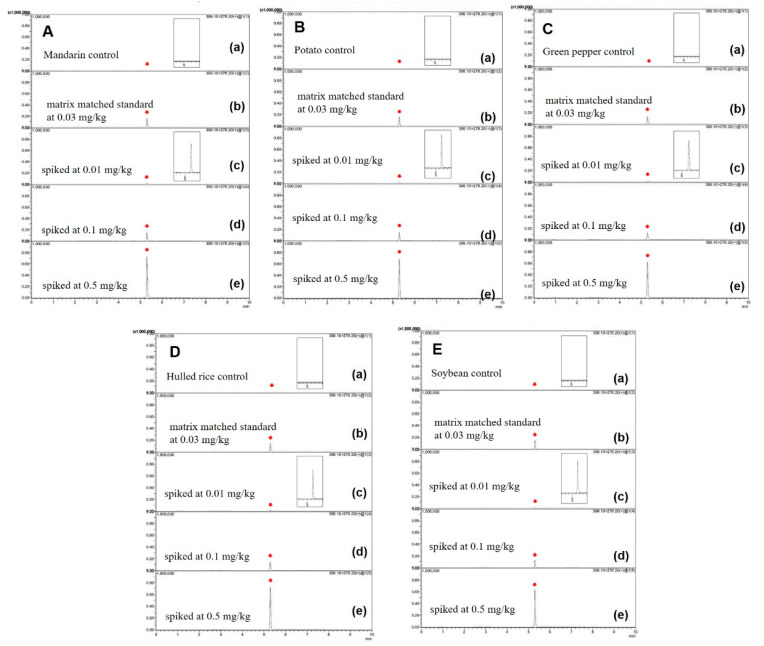
Representative MRM (quantification ion 399 > 278) chromatograms of triflumezopyrim residue in (**A**) mandarin, (**B**) potato, (**C**) green pepper, (**D**) hulled rice, and (**E**) soybean: (**a**) control of each food, (**b**) matrix-matched standard at 0.03 mg/kg, (**c**) spiked at 0.01 mg/kg, (**d**) spiked at 0.1 mg/kg, and (**e**) spiked at 0.5 mg/kg.

Precision was measured by testing the intra-day for repeatability and inter-day for reproducibility. While the intra-day precision was evaluated at the fortified levels (0.01, 0.1, and 0.5 mg/kg) during the same day, the repeatability was assessed using the RSD, which was less than 5.4% for all samples. The inter-day precision was evaluated by analyzing five samples spiked with 10 µg/kg, 100 µg/kg, and 500 µg/kg during 15 consecutive days, and the inter-day RSDs were below 9.3% (Table 3 and Figure 3).

**Figure 4:**
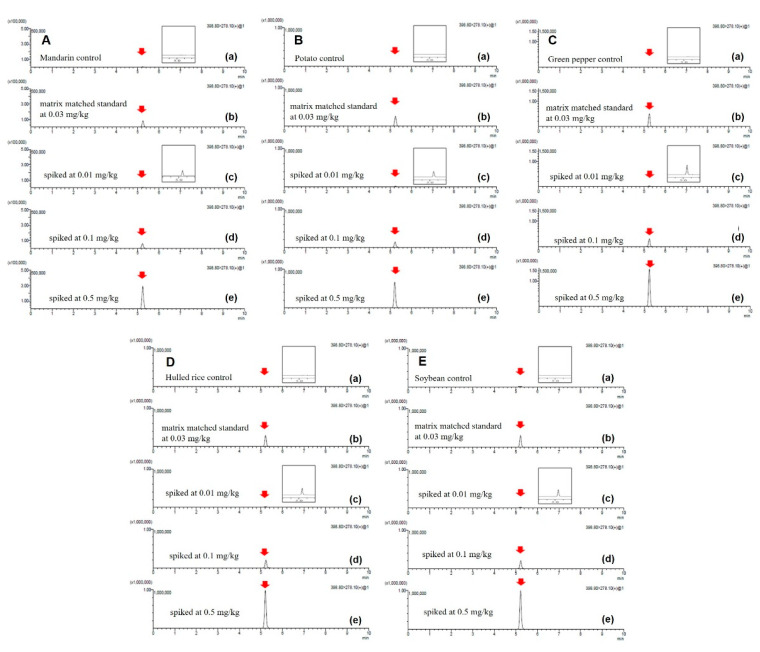
Inter-laboratory comparison of triflumezopyrim residue (quantification ion 399 > 278) in (**A**) mandarin, (**B**) potato, (**C**) green pepper, (**D**) hulled rice, and (**E**) soybean: (**a**) control of each food, (**b**) matrix-matched standard at 0.03 mg/kg, (**c**) spiked at 0.01 mg/kg, (**d**) spiked at 0.1 mg/kg, and (**e**) spiked at 0.5 mg/kg).

As shown in Figure 4, the inter-laboratory comparison by testing the same method in a different laboratory was performed to validate the method (Gwangju Regional Food and Drug Administration). The mean recovery of the triplicates was in the 81.2–108.6% range, with an RSD of less than 9%. The chromatograms for the recovery of the five agricultural products are also acceptable. Therefore, the developed method in this study is in agreement with not only the guidelines of the Codex (CAC/GL 90-2017) but also the guidelines on the standard procedures for preparing the analysis method of MFDS in 2016 [40].

The validation data showed that the developed method represented good linearity, sensitivity, precision, and accuracy for the analysis of triflumezopyrim in agricultural products. Therefore, the developed method is suitable and could be included in the Korean Food Code for enabling the safety management of pesticides and will facilitate the decision-making on the incongruity or congruity of the MRLs. The purpose of this study was the development of an analytical method for the determination of triflumezopyrim residues in various matrices, and the results suggest that the developed method could be successfully applied for the official analysis of triflumezopyrim.

## 4. Conclusions

In this study, a modified QuEChERS method coupled with d-SPE was developed and validated for application to five representative agricultural matrices (green pepper, hulled rice, mandarin, potato, and soybean). The UPLC-MS/MS method for the analysis of triflumezopyrim was optimized and displayed good linearity, with a good correlation coefficient (*R*^2^ = 0.9992–0.9998) in the 0.003–0.30 mg/kg calibration range. The method LOD and LOQ were 0.003 and 0.01 mg/kg, respectively. The mean recovery for triflumezopyrim was in the 89.7 to 104.3% range, and the relative standard deviations were ≤9.8% for intra- (*n* = 5) and inter-day (*n* = 15) precisions at the concentrations of 0.01, 0.1, and 0.5 mg/kg in the five selected food matrices. The proposed method in this study showed satisfactory validation results in terms of linearity, LOD, LOQ, accuracy, and precision. The optimized method represents the first study and is an important tool for the determination of triflumezopyrim in agricultural commodities. The developed method is convenient and could be used for routine analysis for monitoring the presence of triflumezopyrim in commercial agricultural products.

## Figures and Tables

**Table 1:** LC-MS/MS parameters for triflumezopyrim.

Molecular Weight	Exact Mass	Ionization Mode ESI	Retention Time (min)	Precursor Ion (*m*/*z*)	Product Ions (*m*/*z*)	CE ^(1)^ (eV)
398.3	398.09	(+)	5.3	399.10	278.2 ^(2)^	28
					121.25	30
					305.9	26

Note: ^(1)^ Collision energy ^(2)^ Quantification ion.

**Table 2:** A comparison of matrix-matched calibration and solvent calibration.

Matrix	Linear Equation	*R* ^2^	Linear Range (mg/kg)	LOD (mg/kg)	LOQ (mg/kg)	Matrix Effect (%)
Solvent	y = 7,722,939x − 14,567	0.9996	0.003–0.30	0.003	0.01	-
Mandarin	y = 7,296,289x − 19,541	0.9998				−5.5
Potato	y = 7,027,745x − 11,321	0.9992				−9.0
Green pepper	y = 7,191,487x − 18,409	0.9998				−6.9
Hulled rice	y = 6,759,340x − 10,407	0.9997				−12.5
Soybean	y = 7,350,651x − 21,557	0.9998				−4.8

**Table 3:** Validation results of the analytical method for triflumezopyrim at three spiked levels from representative agricultural products.

Matrix	Spiked Level (mg/kg)	Recovery (%) ± RSD (%)		Average Recovery (%) ± RSD (%)
		Intra-Day	Inter-Day	
Mandarin	0.01	102.1 ± 5.4	101.1 ± 5.2	101.4 ± 5.4
	0.1	96.2 ± 1.5	98.3 ± 4.4	97.8 ± 4.0
	0.5	97.1 ± 0.9	98.4 ± 3.8	98.1 ± 3.4
Potato	0.01	103.7 ± 1.0	104.5 ± 1.8	104.3 ± 1.7
	0.1	84.5 ± 5.1	98.1 ± 9.0	94.7 ± 9.8
	0.5	86.6 ± 2.9	90.7 ± 9.3	89.7 ± 8.5
Green pepper	0.01	99.5 ± 1.5	99.8 ± 4.5	99.7 ± 4.0
	0.1	94.7 ± 0.9	98.4 ± 3.0	97.4 ± 3.2
	0.5	85.0 ± 1.4	95.2 ± 8.3	92.6 ± 8.8
Hulled rice	0.01	97.4 ± 2.6	100.6 ± 5.1	99.8 ± 4.9
	0.1	103.7 ± 3.2	98.6 ± 3.8	99.9 ± 4.3
	0.5	99.5 ± 1.2	101.4 ± 2.8	101.0 ± 2.6
Soybean	0.01	103.1 ± 3.9	100.4 ± 6.3	101.1 ± 6.0
	0.1	90.8 ± 1.1	97.5 ± 6.6	95.8 ± 6.6
	0.5	92.1 ± 1.5	91.6 ± 1.6	91.7 ± 1.6

## Data Availability

The data presented in this study are available on request from the corresponding author.

## Supplementary Materials

The following are available online at https://www.mdpi.com/article/10.3390/foods10092090/s1, Table S1: Representative commodities and samples for validation of analytical procedures for pesticide residues, Table S2: Analytical conditions for the determination of triflumezopyrim using UPLC-MS/MS, Table S3: Scheme for preparing matrix-matched calibration standards, Table S4: Effect of NaCl and MgSO4 for extraction efficiency of triflumezopyrim, Table S5: Comparisons of d-SPE adsorbent for purification efficiency of triflumezopyrim, Figure S1: Experimental procedure for the determination of triflumezopyrim residue in food, Figure S2: LC-MS/MS chromatograms for triflumezopyrim standard solution at concentration of 0.1 mg/kg in acetonitrile, Figure S3: LC-MS/MS chromatograms for triflumezopyrim standard curves, Figure S4: Representative MRM (qualification ion 399 > 121) chromatograms of triflumezopyrim residue, Figure S5: Representative MRM (qualification ion 399 > 306) chromatograms of triflumezopyrim residue, Figure S6: Inter-laboratory comparison of triflumezopyrim residue (qualification ion 399 > 121), Figure S7: Inter-laboratory comparison of triflumezopyrim residue (qualification ion 399 > 306).
