# Supplementary material for: Determination of Residual Triflumezopyrim Insecticide in Agricultural Products through a Modified QuEChERS Method
Source: Foods. 2021 Sep 3;10(9):2090. doi: 10.3390/foods10092090 (PMC8472026; doi:10.3390/foods10092090)
Supplement: Supplementary file 1 [file foods-10-02090-s001.zip › foods-1359227-supplementary.pdf]

foods

MDPI

Supplementary Material

Determination of Residual Triflumezopyrim Insecticide in Agricultural Products through a Modified QuEChERS Method

Sung Min Cho [1,2], Han Sol Lee [1], Ji-Su Park [1], Su Jung Lee [1], Hye-Sun Shin [1], Yun mi Chung [3], Ha na Choi [3], Yong-Hyun Jung [1], Jae-Ho Oh [1] and Sang Soon Yun [1,*]

[1] Food Safety Evaluation Department, Pesticide and Veterinary Drug Residues Division, National Insti-tute of Food and Drug Safety Evaluation, Ministry of Food and Drug Safety, Cheongju 28159, Korea; smcho.0101@gmail.com (S.M.C.); leehs3029@korea.kr (H.S.L.); jeesoo0320@korea.kr (J.-S.P.); bplsj@korea.kr (S.J.L.); hyesun0714@korea.kr (H.-S.S.); jyh311@korea.kr (Y.-H.J.); chopin68@korea.kr (J.-H.O.)

[2] Department of Integrated Biomedical and Life Science, Graduate School, Korea University, Seoul 02841, Korea

[3] Hazardous Substances Analysis Division, Gwangju Regional Food and Drug Administration, Gwangju 61012, Korea; gd96@korea.kr (Y.m.C.); chlgkskgg@korea.kr (H.n.C.)

*Correspondence: yss0520@korea.kr; Tel.: +82-43-719-4211

Commodity Group	Common Properties	Commodity Class	Representative Species
I	High water and chlorophyll content	Leafy vegetables<br>Brassica leafy vegetables<br>Legume vegetables	spinach or lettuce<br>broccoli, cabbage, kale<br>green beans, green pepper
II	High water and low or no chlorophyll content	Pome fruits<br>Stone fruits<br>Berries<br>Small fruits<br>Fruiting vegetables<br>Root vegetables	apple, pear<br>peach, cherry<br>Strawberry<br>grape,<br>tomato, bell pepper, melon<br>mushroom<br>potato, carrot, parsley
III	High acid content	Citrus fruits	Orange, lemon, mandarin
IV	High sugar content		raisins, dates
V	High oil or fat	Oil seeds<br>Nuts	avocado, sunflower seed<br>walnut, pecan nut, pistachios, soybean
V	Dry materials	Cereals	wheat, rice or maize grains, hulled rice
		Cereal products	wheat bran, wheat floor

Table S1. Representative commodities and samples for validation of analytical procedures for pesticide residues (modified from [23]).

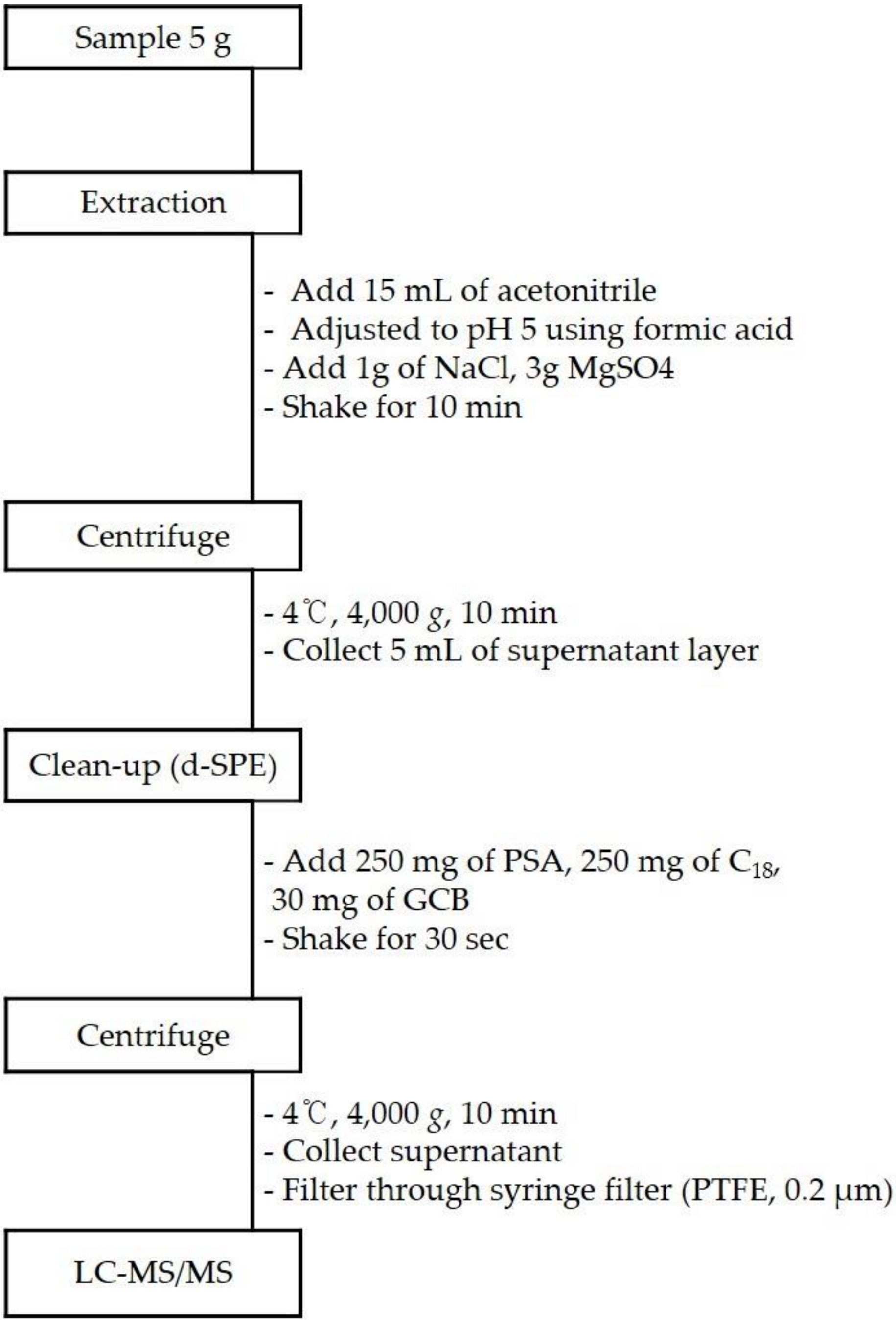

Figure S1. Experimental procedure for the determination of triflumezopyrim residue in food.

Table S2. Analytical conditions for the determination of triflumezopyrim using UPLC-MS/MS.

Condition	Content		
Instrument	LC: Nexera X2 UPLC(Shimadzu, Kyoto, Japan)<br>MS/MS: LCMS-8060(Shimadzu, Kyoto, Japan)		
**Chromatographic separation**			
Column	XBridge C_{18} (2.1 mm I.D. × 100 mm L., 3.5 μm)		
Flow rate	0.3 mL/min		
Injection volume	2 μL		
Oven temp.	40℃		
Mobile phase	A: 0.1% formic acid in acetonitrile<br>B: 0.1% formic acid in water		
- Gradient	Time(min)	A(%)	B(%)
	0.0	10	90
	1.0	30	70
	4.0	70	30
	7.0	30	70
	8.0	10	90
	10.0	10	90
**MS/MS condition**			
Interface temp.	150℃		
Heating block temp.	400℃		
Desolvation line temp.	250℃		
Heating gas flow	10.0 L/min		
Nebulizer gas flow	3.0 L/min		

Table S3. Scheme for preparing matrix-matched calibration standards.

Calibration levels		Matrix-matched calibration standards(μg/mL)						
		0.003	0.006	0.012	0.03	0.06	0.15	0.3
Sample extract		900 μL	900 μL	900 μL	900 μL	900 μL	900 μL	900 μL
MMS* (μg/mL)	3	-	-	-	-	-	-	100 μL
	1.5	-	-	-	-	-	100 μL	-
	0.6	-	-	-	-	100 μL	-	-
	0.3	-	-	-	100 μL	-	-	-
	0.12	-	-	100 μL	-	-	-	-
	0.06	-	100 μL	-	-	-	-	-
	0.03	100 μL	-	-	-	-	-	-
Total volume		1,000 μL	1,000 μL	1,000 μL	1,000 μL	1,000 μL	1,000 μL	1,000 μL

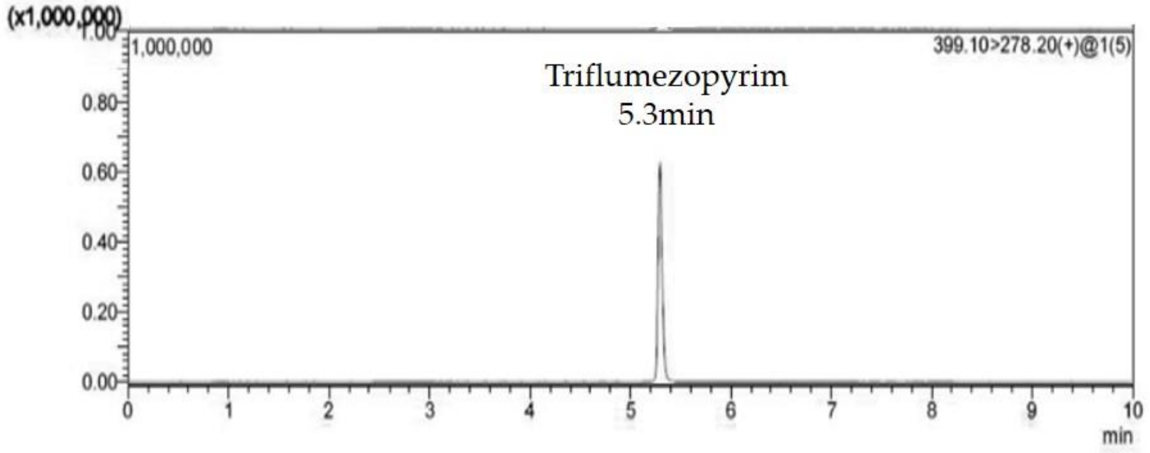

Figure S2. LC-MS/MS chromatograms for triflumezopyrim standard solution at concentration of 0.1 mg/kg in acetonitrile.

Table S4. Effect of NaCl and $MgSO_4$ for extraction efficiency of triflumezopyrim.

	Recovery ± RSD[a](%)		
	NaCl 0 g	NaCl 1 g	NaCl 2 g
Mandarin	79.5 ± 2.4	99.8 ± 1.2	102.9 ± 0.8
Soybean	88.2 ± 3.2	93.2 ± 3.6	90.1 ± 0.8
	Recovery ± RSD[a](%)		
	$MgSO_4$ 0 g	$MgSO_4$ 1 g	$MgSO_4$ 3 g
Mandarin	96.5 ± 1.5	97.8 ± 1.4	101.3 ± 3.6
Soybean	95.4 ± 2.4	99.5 ± 2.1	100.1 ± 0.6

Table S5. Comparisons of d-SPE adsorbent for purification efficiency of triflumezopyrim.

Compound	Recovery ± RSD[a](%)
150 mg $MgSO_4$, 50 mg PSA, 50 mg C_{18}, 5 mg GCB	26.5 ± 7.7
150 mg $MgSO_4$	51.5 ± 4.2
50 mg PSA	93.9 ± 0.1
50 mg C_{18}	97.0 ± 0.7
5 mg GCB	22.7 ± 3.8

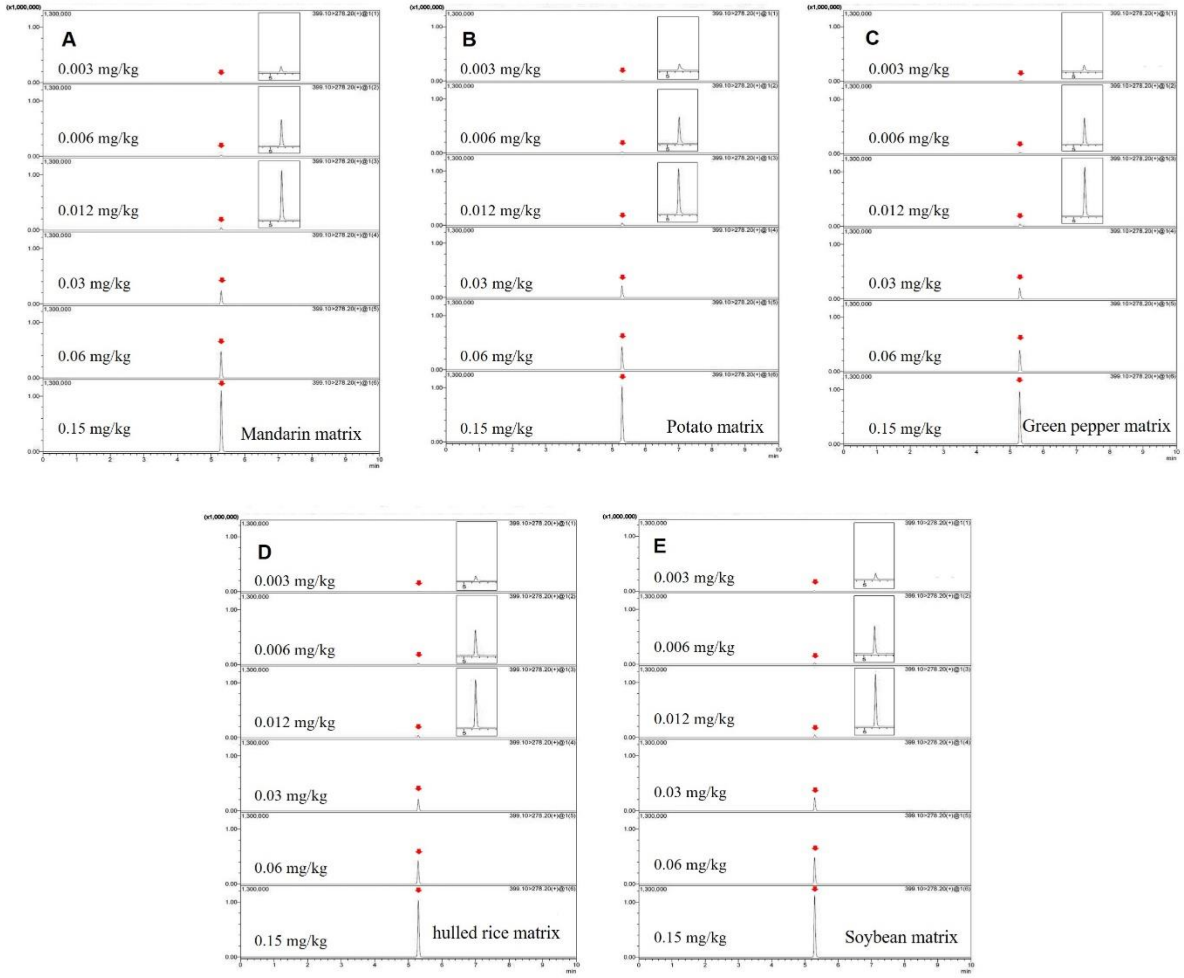

Figure S3. LC-MS/MS chromatograms for triflumezopyrim standard curves: (A) mandarin, (B) potato, (C) green pepper, (D) hulled rice, and (E) soybean.

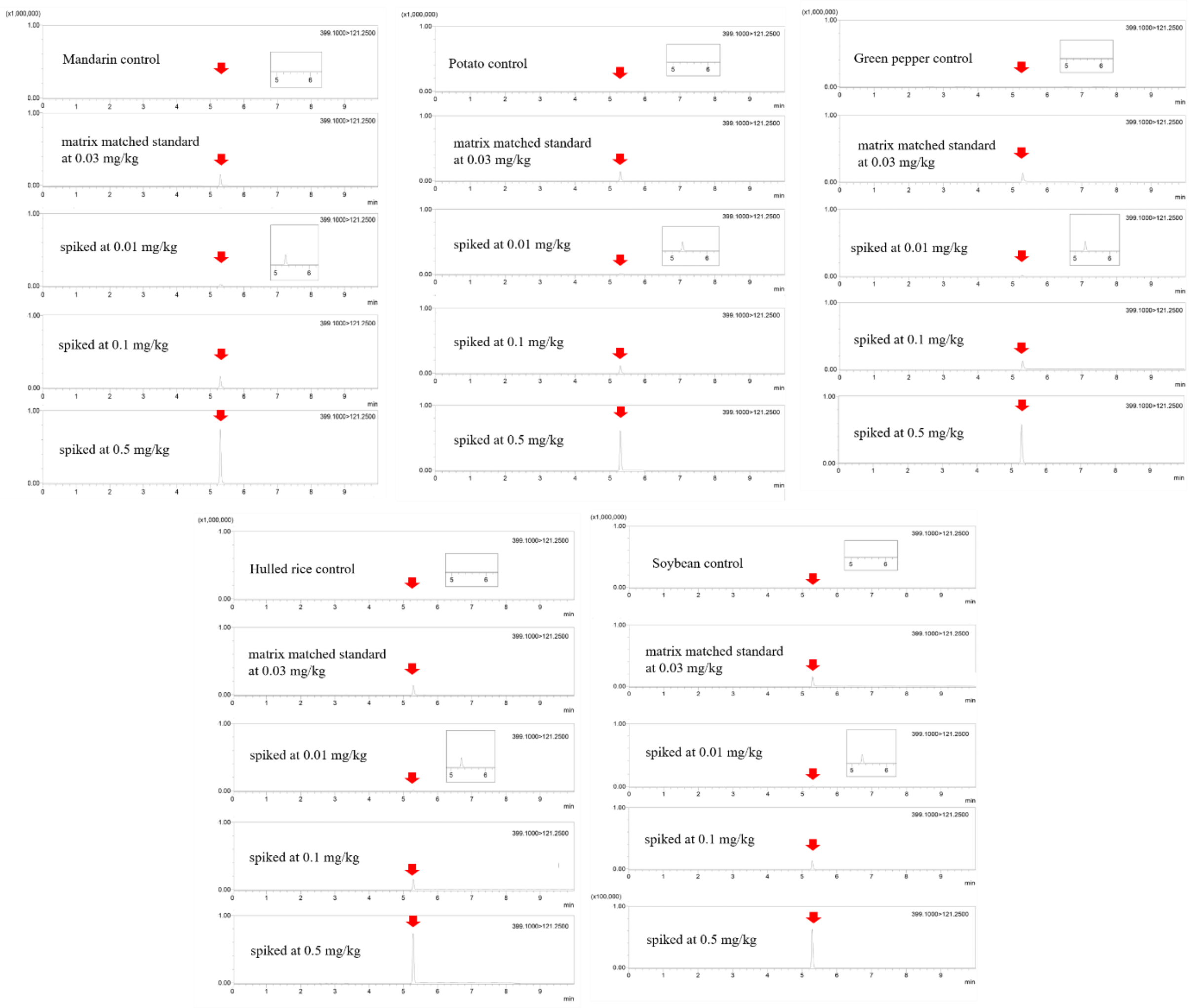

Figure S4. Representative MRM (qualification ion 399>121) chromatograms of triflumezopyrim residue in (A) mandarin, (B) potato, (C) green pepper, (D) hulled rice, and (E) soybean: (a) control of each food, (b) matrix matched standard at 0.03 mg/kg, (c) spiked at 0.01 mg/kg, (d) spiked at 0.1 mg/kg and (e) spiked at 0.5 mg/kg.

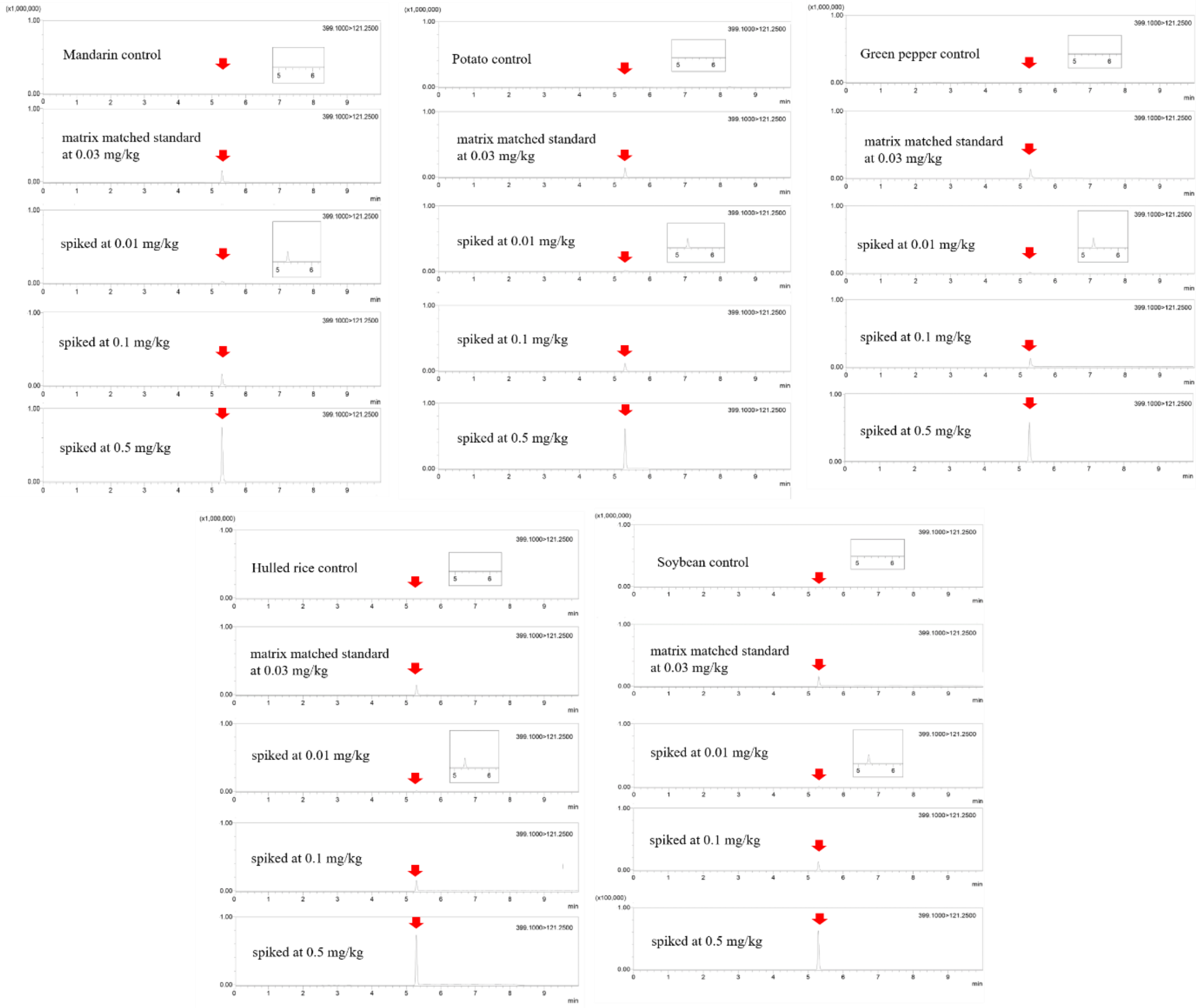

Figure S5. Representative MRM (qualification ion 399>306) chromatograms of triflumezopyrim residue in (A) mandarin, (B) potato, (C) green pepper, (D) hulled rice, and (E) soybean: (a) control of each food, (b) matrix matched standard at 0.03 mg/kg, (c) spiked at 0.01 mg/kg, (d) spiked at 0.1 mg/kg and (e) spiked at 0.5 mg/kg.

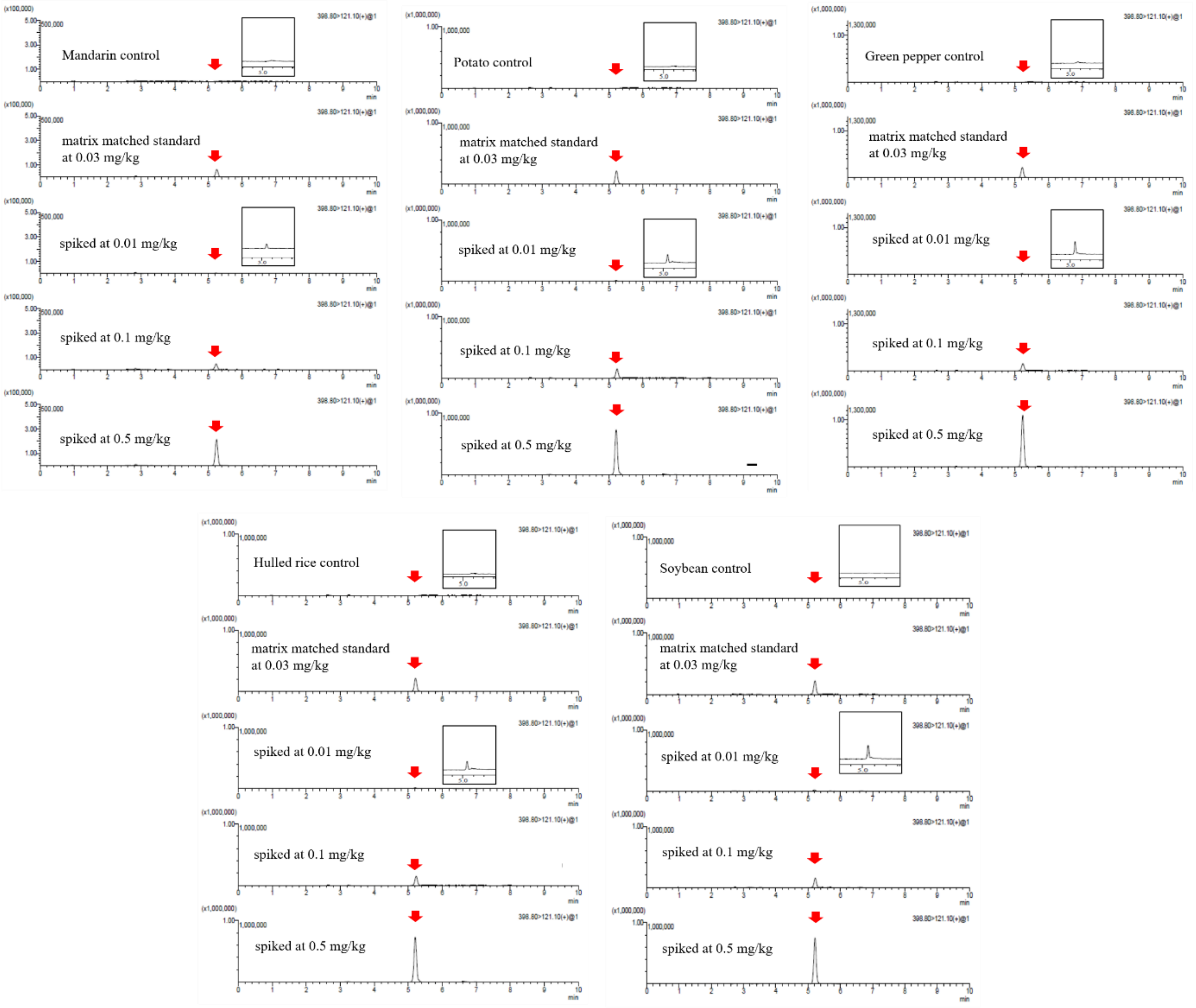

Figure S6. Inter-laboratory comparison of triflumezopyrim residue (qualification ion 399>121) in (A) mandarin, (B) potato, (C) green pepper, (D) hulled rice, and (E) soybean: (a) control of each food, (b) matrix matched standard at 0.03 mg/kg, (c) spiked at 0.01 mg/kg, (d) spiked at 0.1 mg/kg and (e) spiked at 0.5 mg/kg).

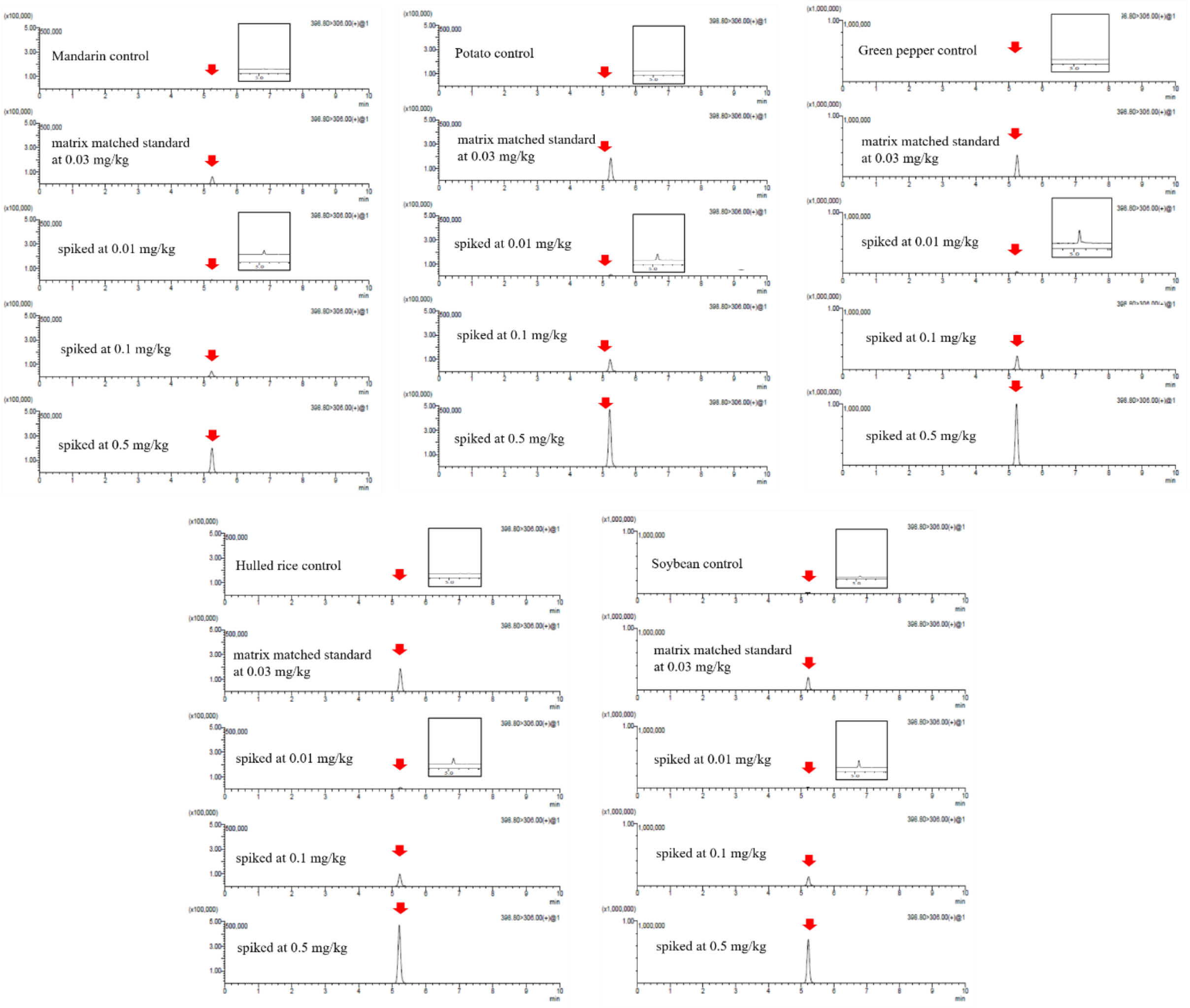

Figure S7. Inter-laboratory comparison of triflumezopyrim residue (qualification ion 399>306) in (A) mandarin, (B) potato, (C) green pepper, (D) hulled rice, and (E) soybean: (a) control of each food, (b) matrix matched standard at 0.03 mg/kg, (c) spiked at 0.01 mg/kg, (d) spiked at 0.1 mg/kg and (e) spiked at 0.5 mg/kg).
